# Associations among grazing behavior, emotional eating, and psychological well-being in university students: A cross-sectional study

**DOI:** 10.1097/MD.0000000000050909

**Published:** 2026-09-25

**Authors:** Vesile Adigüzel, Mehmet Akif Cansiz, Muhammet Mutlu Özbek, Handan Işiklar, Ekin Atay

**Affiliations:** a Department of Nursing, Faculty of Health Sciences, Psychiatric Nursing, Yalova University, Yalova, Turkey; b Department of Child and Adolescent Psychiatry, Faculty of Medicine, Yalova University, Yalova, Turkey; c Department of Internal Medicine, Faculty of Medicine, Yalova University, Yalova, Turkey; d Department of Psychiatry, Faculty of Medicine, Internal Medicine Sciences, Manisa Celal Bayar University, Manisa, Turkey.

**Keywords:** emotional eating, grazing behavior, psychological well-being

## Abstract

This study aimed to examine the associations among grazing behavior, emotional eating, and psychological well-being and to identify variables statistically associated with emotional eating among university students. A descriptive, cross-sectional, and correlational design was employed. Data were collected from 540 students between May and July 2025 through face-to-face interviews using a personal information form, the Grazing Questionnaire, the Emotional Eating Scale, and the Psychological Well-Being Scale. Descriptive statistics, including frequencies, percentages, means, and standard deviations, were calculated. Sociodemographic differences were analyzed using independent samples *t*-tests and 1-way analysis of variance, while relationships between scales were examined with Pearson correlation analysis. Multiple linear regression analysis was conducted to identify variables statistically associated with emotional eating. Of the participants, 71.9% (n = 388) were female. On average, students reported low levels of grazing behavior, exhibited emotional eating behaviors, and demonstrated moderate levels of psychological well-being according to scale scores. A significant positive correlation was found between grazing behavior and emotional eating, whereas psychological well-being was significantly negatively correlated with emotional eating. In the regression model, grazing behavior showed the strongest positive association with emotional eating. The model including grazing behavior, psychological well-being, body mass index, and gender explained 36.6% of the variance in total emotional eating scores. These findings suggest that university-based counseling programs, public health initiatives, and psychological support interventions may help address grazing and emotional eating behaviors among students.

## 1. Introduction

University years represent a period in which individuals move away from the family environment, gain greater independence, and experience multiple academic, social, and psychological changes. During this process, students may face various challenges such as financial difficulties, academic stress, shifts in social relationships, and adjustment issues, all of which can strain their psychological well-being.^[1]^ For young adults who are struggling to meet both biological and sociocultural needs, managing psychological demands is equally critical. Fluctuations in emotional states can markedly influence eating behaviors: feelings of anger, anxiety, or pressure may be associated with overeating in some individuals, whereas heightened excitement or stress may suppress appetite and lead to insufficient intake.^[2,3]^ Studies conducted among university students have shown that variables such as academic stress, self-perception, and depression may play mediating or moderating roles in the relationship between emotions and eating.^[4,5]^

Emotional eating is a pattern of food intake that occurs in response to emotional states rather than physical hunger. It often arises not only from negative emotions such as stress, sadness, or distress, but also from positive emotions.^[6]^ Thus, it is considered distinct from routine eating behaviors in that it serves to regulate or cope with emotional experiences rather than to satisfy physiological needs.^[7]^ Research has shown that positive emotions such as happiness can enhance the pleasure derived from eating, thereby encouraging healthier food choices and more balanced consumption.^[8]^ In contrast, negative emotions such as stress, anxiety, depression, and anger have been associated with increased consumption of energy-dense foods, contributing to unhealthy dietary patterns. This effect appears to be particularly pronounced among university students, where it also interacts with body mass index (BMI) and lifestyle factors.^[2,3]^ Moreover, individuals with emotional eating tendencies have been observed to frequently turn to high-calorie, unhealthy foods and to sustain these eating habits through grazing behavior.^[9]^

Grazing, one of the unhealthy eating behaviors, is characterized by the repetitive and unplanned consumption of small amounts of food. This pattern typically occurs outside of regular meals or snacks, is not driven by hunger or satiety cues, and is regarded as a maladaptive form of eating.^[10,11]^ Although grazing shares certain features with binge eating and emotional eating (such as emotional triggers or a sense of loss of control) binge eating is usually defined by large, episodic, and distressing episodes with clear psychological consequences.^[12]^ Some studies have shown that grazing behavior is common among student populations.^[13,14]^

Psychological well-being is a multidimensional construct encompassing positive affect, life satisfaction, autonomy, self-acceptance, purpose in life, environmental mastery, and positive social relationships.^[15]^ It refers not only to the absence of psychological problems but also to the presence of positive emotions and cognitive evaluations such as optimism and a sense of meaning in life. Higher levels of psychological well-being are associated with better health outcomes and greater life satisfaction.^[16]^ Research among university students has consistently demonstrated a negative association between psychological well-being and emotional eating.^[1,17,18]^ From an emotion regulation perspective, emotional eating and grazing behavior may represent maladaptive behavioral responses to negative affect, stress, or psychological discomfort. When individuals have difficulty identifying, tolerating, or modifying distressing emotional states, eating may provide an immediately accessible but short-term means of reducing emotional arousal. Cognitive emotion regulation strategies such as rumination, self-blame, and catastrophizing may further intensify psychological distress, whereas adaptive strategies may reduce reliance on potentially harmful behavioral responses. Evidence from student populations and other behavioral contexts indicates that impulsivity, cognitive emotion regulation, emotional functioning, and broader mental health factors may be associated with vulnerability to maladaptive behavioral tendencies.^[19–22]^ Stress and coping resources may also contribute to this process. University students frequently encounter academic demands, financial concerns, changing social relationships, and adaptation to independent living. When perceived demands exceed available coping resources, individuals may resort to emotion-focused or avoidant behaviors, including unplanned or repetitive eating. In contrast, self-efficacy (the perceived ability to manage challenging situations) and adaptive coping strategies may support more effective emotional and behavioral regulation. Previous research conducted in different health and student populations has demonstrated that self-efficacy and coping styles may be associated with stress-related outcomes and broader mental health indicators.^[20,23]^ Although these mechanisms have not been directly examined in relation to grazing behavior in the present study, psychological well-being may be conceptualized as a broader protective resource reflecting positive functioning, self-acceptance, purpose in life, and environmental mastery. Lower psychological well-being may increase vulnerability to eating-related coping, whereas higher well-being may facilitate more adaptive responses to emotional distress. Although previous studies have examined emotional eating in relation to stress, BMI, and psychological distress, fewer studies have focused on grazing behavior among university students. Moreover, the combined relationship among grazing behavior, emotional eating, and psychological well-being has not been sufficiently addressed. This gap is important because grazing may represent a repetitive and less visible form of dysregulated eating that can accompany emotional eating tendencies, particularly in young adults experiencing psychological strain.

Therefore, this study aimed to examine the associations among grazing behavior, emotional eating, and psychological well-being and to identify factors statistically associated with emotional eating among university students. Based on the emotion regulation perspective and existing literature, the following hypotheses were proposed: (H1) grazing behavior would be positively associated with emotional eating; (H2) psychological well-being would be negatively associated with emotional eating; (H3) psychological well-being would be negatively associated with grazing behavior; and (H4) grazing behavior, psychological well-being, BMI, and gender would be statistically associated with emotional eating in the regression model.

## 2. Methods

### 2.1. Study design

This research was designed as a descriptive, cross-sectional, and correlational study. The reporting of this study was guided by the Strengthening the Reporting of Observational Studies in Epidemiology statement.

### 2.2. Population and sample

The study population consisted of students actively enrolled at X University. Participants were recruited using a non-probability convenience sampling method. Students who were present on campus during the data collection period, met the inclusion criteria, and voluntarily agreed to participate were included in the study. The sample size was calculated by the researchers using the Power Analysis & Sample Size (version 11.0.10; NCSS, LLC) statistical software. For a correlation test, the required sample size was determined to be 300 (power = 80%, alpha = 0.05). Considering the possibility of incomplete responses and to increase the precision of the estimates, data were collected from 540 students.

### 2.3. Setting and data collection period

Data were collected face-to-face from actively enrolled university students between May and July 2025.

### 2.4. Data collection tools

The data were obtained using a Personal Information Form, the Grazing Questionnaire, the Emotional Eating Scale, and the Psychological Well-Being Scale (PWBS).

Personal Information Form: a 7-item form developed by the researchers to collect demographic data, including gender, height, weight, year of study, and place of residence.
Grazing Questionnaire: developed by Lane and Szabo (2013) to assess grazing-style eating, this 8-item scale uses a 5-point Likert format (0 = “Never,” 4 = “Always”). Five items measure grazing behavior (e.g., “Do you find yourself constantly eating small amounts of food?”), while 3 items assess loss of control (e.g., “Do you ever feel unable to stop snacking between meals?”). Higher scores indicate more severe grazing behavior. Scores range from 0 to 32. The scale was adapted into Turkish by Akkaya and Yilmaz (2023).^[14]^
Emotional Eating Scale: originally developed by Garaulet et al (2012) and adapted into Turkish by Arslantaş et al (2019), this 10-item scale includes 3 subdimensions: disinhibition (inability to control eating urges), type of food, and guilt. Items are rated on a 4-point Likert scale (0 = “Never,” 1 = “Sometimes,” 2 = “Often,” 3 = “Always”).^[24]^ The total score ranges from 0 to 30, with higher scores indicating higher levels of emotional eating. Scores are categorized as follows: 0 to 5 = not an emotional eater; 6 to 10 = low level; 11 to 20 = emotional eater; 21 to 30 = very emotional eater.
PWBS: developed by Diener et al (2010) as a brief and comprehensive measure of psychological well-being and adapted into Turkish by Telef (2011).^[25]^ Cronbach alpha for the Turkish version was reported as.80, and test–retest reliability indicated a strong, positive correlation (*r* = .86, *P* < .01). The scale consists of 8 items scored on a 7-point Likert scale ranging from 1 (strongly disagree) to 7 (strongly agree), yielding a total score between 8 and 56. Higher scores reflect greater psychological resources and strengths.

### 2.5. Data analysis

Statistical analyses were conducted using International Business Machines Corporation Statistical Package for the Social Sciences Statistics for Windows, version 23. Before conducting parametric analyses, the normality of the data was assessed visually using Q–Q plots and statistically using skewness and kurtosis values. Skewness and kurtosis values within the range of ± 3 were considered acceptable for normal distribution. Descriptive statistics, including frequencies, percentages, means, and standard deviations, were reported. Cronbach alpha coefficients were calculated to evaluate the internal consistency of the scales. Independent samples *t*-tests and 1-way analysis of variance were used to compare scale scores according to sociodemographic variables, while Pearson correlation analysis was conducted to examine the associations among numerical variables.

Prior to linear regression analysis, the relevant assumptions were examined. The normality of residuals was assessed using histogram and normal probability plots. Linearity and homoscedasticity were evaluated through scatterplots of standardized residuals and predicted values. Multicollinearity was examined using tolerance and variance inflation factor values. No serious violations of regression assumptions were observed. A multiple linear regression analysis was conducted to identify variables statistically associated with emotional eating. In all statistical analyses, the level of significance was accepted as *P* < .05.

### 2.6. Ethical considerations

The study was conducted in accordance with the Declaration of Helsinki. Ethical approval was obtained from the Non-Interventional Research Ethics Committee of Yalova University (Decision no: 2025/153, dated 23.05.2025). Institutional permission was also granted (E-91559680-605-2500037002). Written and verbal informed consent was obtained from all participants.

## 3. Results

A total of 540 university students participated in the study. The mean body weight of participants was 63.05 ± 12.75 kg, mean height was 167.34 ± 8.37 cm, and mean BMI was 22.42 ± 3.65 kg/m^2^. According to BMI categories, 8.0% were underweight, 71.7% were normal weight, 16.5% were overweight, 3.5% were obese, and 0.4% were morbidly obese. Among the participants, 71.9% (n = 388) were female; 34.1% (n = 184) reported smoking, and 17.8% (n = 96) reported alcohol use. Regarding place of residence, 74.4% (n = 402) lived in dormitories, 10.6% (n = 57) with family, and 15.0% (n = 81) alone (Table 1).

**Table 1:** Sociodemographic characteristics of the participants.

		Sample (n = 540)
Weight, kg, mean ± SD		63.05 ± 12.75
Height, cm, mean ± SD		167.34 ± 8.37
BMI, kg/m^2^, mean ± SD		22.42 ± 3.65
BMI categories, n (%)	Underweight	43 (8.0)
	Normal	387 (71.7)
	Overweight	89 (16.5)
	Obese	19 (3.5)
	Morbidly obese	2 (0.4)
Year of school, n (%)	1 yr	156 (28.9)
	2 yr	173 (32.0)
	3 yr	140 (25.9)
	4 yr	71 (13.1)
Gender, female, n (%)		388 (71.9)
Smoking, n (%)		184 (34.1)
Alcohol, n (%)		96 (17.8)
Place of residence, n (%)	Dormitories	402 (74.4)
	Family	57 (10.6)
	Alone	81 (15.0)

BMI = body mass index, n = number of participants, SD = standard deviation.

The mean total score of the Grazing Questionnaire was 12.03 ± 5.56, with subscale means of 8.07 ± 3.66 for grazing behavior and 3.96 ± 2.67 for loss of control. The mean total score of the Emotional Eating Scale was 11.58 ± 6.04; subscale means were 6.47 ± 3.93 for disinhibition, 2.81 ± 1.31 for type of food, and 2.29 ± 1.69 for guilt. The mean total score of the PWBS was 36.97 ± 9.12.

Table 2 presents the correlation results among the scales. Psychological well-being was moderately and negatively correlated with the total score of emotional eating (*r* = −0.244, *P* < .001), the disinhibition subscale (*r* = −0.241, *P* < .001), and the guilt subscale (*r* = −0.247, *P* < .001). It was also negatively correlated, albeit weakly, with the loss of control subscale of the Grazing Questionnaire (*r* = −0.149, *P* < .001) and with the total grazing score (*r* = −0.086, *P* = .046). No significant associations were found between psychological well-being and the type of food subscale of the Emotional Eating Scale (*r* = −0.083, *P* = .055) or the grazing behavior subscale of the Grazing Questionnaire (*r* = −0.022, *P* = .614).

**Table 2:** Correlation among scales.

		Grazing loss of control	Grazing behavior	Grazing questionnaire total	Emotional eating disinhibation	Emotional eating type of food	Emotional eating guilt	Emotional eating total
Grazing loss of control	Pearson *r*	-						
	*P*	-						
Grazing behavior	Pearson *r*	0.527***	-					
	*P*	< .001	-					
Grazing questionnaire total	Pearson *r*	0.828***	0.913***	-				
	*P*	< .001	< .001	-				
Emotional eating disinhibation	Pearson *r*	0.503***	0.341***	0.467***	-			
	*P*	< .001	< .001	< .001	-			
Emotional eating type of food	Pearson *r*	0.324***	0.245***	0.317***	0.580***	-		
	*P*	< .001	< .001	< .001	< .001	-		
Emotional eating guilt	Pearson *r*	0.513***	0.412***	0.518***	0.655***	0.366***	-	
	*P*	< .001	< .001	< .001	< .001	< .001	-	
Emotional eating total	Pearson *r*	0.542***	0.391***	0.519***	0.962***	0.698***	0.787***	-
	*P*	< .001	< .001	< .001	< .001	< .001	< .001	-
Psychological well-being	Pearson *r*	−0.149***	−0.022	−0.086*	−0.241***	−0.083	−0.247***	−0.244
	*P*	< .001	.614	.046	< .001	.055	< .001	< .001

* *P* < .05.

** *P* < .01.

*** *P* < .001.

A multiple linear regression model was conducted to identify variables statistically associated with total emotional eating scores. The model was statistically significant (*F* = 77.4, *P* < .001) and explained 36.6% of the variance in emotional eating scores (*R*^2^ = 0.366), indicating a moderate explanatory contribution. As shown in Table 3, grazing behavior showed the strongest positive association with emotional eating (*B* = 0.533, *β* = 0.491, 95% confidence interval [CI]: 0.460–0.607, *P* < .001). BMI was also positively associated with emotional eating (*B* = 0.375, *β* = 0.227, 95% CI: 0.262–0.488, *P* < .001). Psychological well-being showed a negative association with emotional eating (*B* = −0.152, *β* = −0.229, 95% CI: −0.197–−0.107, *P* < .001). Gender was also statistically associated with emotional eating, indicating that male students had lower emotional eating scores than female students (*B* = −1.357, *β* = −0.225, 95% CI: −2.273–−0.441, *P* = .004).

**Table 3:** Variables statistically associated with emotional eating in the multiple linear regression model.

Predictor	Estimate	95% CI			*t*	*P*	Standard estimate	95% CI	
		SE	Lower	Upper				Lower	Upper
Intercept*	2.755	1.5689	−0.327	5.837	1.76	.08			
Grazing	0.533	0.0376	0.460	0.607	14.20	< .001	0.491	0.423	0.5589
BMI	0.375	0.0573	0.262	0.488	6.54	< .001	0.227	0.159	0.2948
PWBS	−0.152	0.0231	−0.197	−0.107	−6.58	< .001	−0.229	−0.298	−0.1609
Gender 1: Female, 2: Male:									
2–1	−1.357	0.4663	−2.273	−0.441	−2.91	.004	−0.225	−0.376	−0.0730

BMI = body mass index, CI = confidence interval, PWBS = Psychological Well-Being Scale, SE = standard error.

* Represents reference level.

## 4. Discussion

The term grazing is generally defined as the repetitive and unstructured consumption of small amounts of food, occurring independently of hunger or satiety cues.^[26]^ Previous studies have shown that individuals with higher levels of grazing behavior often report elevated levels of depression and anxiety, and similar to binge eating, grazing may also serve as a maladaptive strategy for regulating negative affect.^[27,28]^ In our study, psychological well-being was negatively associated with grazing behavior, albeit at a low level. This finding suggests that higher psychological well-being is associated with lower grazing tendencies, highlighting a possible protective role of well-being against maladaptive eating patterns. To our knowledge, no prior studies have directly addressed this relationship, indicating the need for further research to identify risk groups and influencing factors related to grazing.

Our findings showed that grazing behavior had the strongest positive association with emotional eating in the regression model. This finding suggests that grazing behavior and emotional eating may be closely related among university students; however, given the cross-sectional design of the study, this association should not be interpreted as causal. The association between grazing behavior and emotional eating may be interpreted within an emotion regulation framework. Grazing may function as a repetitive, easily accessible behavioral strategy for managing unpleasant emotions or psychological tension. Unlike discrete binge-eating episodes, grazing may occur in a more continuous and less noticeable manner throughout the day, which may make it difficult for students to recognize it as a maladaptive eating pattern. This may be particularly relevant in university settings, where irregular schedules, academic stress, and easy access to snacks may facilitate unplanned eating. Therefore, the observed association between grazing and emotional eating may reflect a broader pattern of dysregulated emotion-related eating rather than a simple dietary habit.^[29,30]^

Emotional eating is widely recognized as a behavior that occurs in response to emotional states rather than physiological hunger and may function as a maladaptive strategy for coping with distress.^[1,31]^ In the present study, psychological well-being was negatively associated with emotional eating, suggesting that students with lower levels of positive functioning, self-acceptance, life purpose, and perceived competence may be more likely to use eating as an emotion-focused coping behavior. This finding is consistent with previous studies showing that stress, academic concerns, depressive symptoms, and psychological distress are associated with emotional eating among university students.^[32–38]^ However, due to the cross-sectional design of the study, the direction of this association cannot be determined. It is also possible that repeated emotional or unplanned eating behaviors may contribute to lower psychological well-being over time. Therefore, longitudinal studies are needed to clarify the temporal and potential bidirectional nature of these relationships.

In the regression model, grazing behavior, BMI, psychological well-being, and gender were statistically associated with emotional eating and together explained 36.6% of the variance. This level of explained variance suggests a moderate explanatory contribution, indicating that these variables are relevant to emotional eating among university students. However, a substantial proportion of variance remained unexplained, suggesting that other factors not assessed in this study, such as stress, depressive symptoms, anxiety, socioeconomic status, sleep quality, physical activity, and dietary habits, may also contribute to emotional eating. Supporting our results, prior research has shown that food preferences during emotional states differ: individuals in positive moods tend to prefer savory foods such as pizza, steak, or stew, whereas those in negative moods are more likely to consume desserts such as ice cream, cookies, or sweets.^[39]^ In our study, BMI was positively associated with emotional eating, suggesting that students with higher BMI tended to report higher emotional eating scores. Similarly, Choi (2020) found that emotional eating triggered by negative emotions was linked to ineffective weight control.^[30]^ Gender was also statistically associated with emotional eating; male students reported lower emotional eating scores than female students. This aligns with previous research indicating that women are more vulnerable to hormonal changes, stress, and emotional fluctuations, and that emotional eating is more prevalent among female students.^[1,5,40–42]^

From a practical perspective, these findings suggest that university-based health promotion programs should not focus solely on dietary education but should also include components addressing emotion regulation, stress management, psychological well-being, and awareness of unplanned repetitive eating patterns. Screening students for emotional eating and grazing tendencies may help identify individuals who could benefit from early counseling or psychological support.

## 5. Limitations

This study has several limitations. First, the cross-sectional and correlational design does not allow for causal or temporal inferences among grazing behavior, emotional eating, and psychological well-being. Second, the data were collected from students at a single university using a non-probability convenience sampling method, and the sample was predominantly female, which may limit the generalizability of the findings. Third, all measures were based on self-report instruments, which may have introduced recall bias, response bias, and social desirability bias. Fourth, although BMI and gender were included in the regression model, several potentially relevant confounding variables, such as perceived stress, depression, anxiety, sleep quality, socioeconomic status, dietary habits, physical activity, and other lifestyle factors, were not assessed or controlled. Finally, participants may have reported problematic eating behaviors without having received a formal clinical diagnosis. Future studies using longitudinal designs, more representative samples, and broader psychosocial and lifestyle variables are needed to clarify the direction and mechanisms of these associations.

## 6. Conclusion

This study found that grazing behavior, psychological well-being, BMI, and gender were statistically associated with emotional eating among university students. Grazing behavior showed the strongest positive association with emotional eating, whereas psychological well-being was negatively associated with emotional eating. These findings suggest that emotional eating among university students should be considered within a broader psychosocial and behavioral framework. University-based counseling, psychological support, and health promotion programs may be useful for addressing dysregulated eating patterns and supporting psychological well-being among students.

## Acknowledgments

The authors would like to thank all students who participated in this study.

## Author contributions

**Conceptualization:** Vesile Adigüzel, Mehmet Akif Cansiz.

**Data curation:** Vesile Adigüzel, Handan Işiklar.

**Formal analysis:** Vesile Adigüzel, Handan Işiklar.

**Funding acquisition:** Vesile Adigüzel.

**Investigation:** Vesile Adigüzel, Ekin Atay

**Methodology:** Vesile Adigüzel, Mehmet Akif Cansiz, Muhammet Mutlu Özbek, Ekin Atay.

**Project administration:** Vesile Adigüzel.

**Resources:** Vesile Adigüzel.

**Software:** Ekin Atay.

**Supervision:** Vesile Adigüzel, Muhammet Mutlu Özbek.

**Validation:** Vesile Adigüzel, Mehmet Akif Cansiz.

**Writing – original draft:** Vesile Adigüzel, Mehmet Akif Cansiz.

**Writing – review & editing:** Vesile Adigüzel, Muhammet Mutlu Özbek.
